# Continued Trauma: A Thematic Analysis of the Asylum-Seeking Experience Under the Migrant Protection Protocols

**DOI:** 10.1089/heq.2020.0144

**Published:** 2021-04-30

**Authors:** Madeleine C. Silverstein, Rebecca F.P. Long, Elizabeth Burner, Parveen Parmar, Todd W. Schneberk

**Affiliations:** ^1^ Keck School of Medicine of University of Southern California, Los Angeles, California, USA.; ^2^ Divisions of Research, Department of Emergency Medicine, LAC+USC Medical Center, Keck School of Medicine of University of Southern California, Los Angeles, California, USA.; ^3^ Divisions of Global Emergency Medicine, Department of Emergency Medicine, LAC+USC Medical Center, Keck School of Medicine of University of Southern California, Los Angeles, California, USA.; ^4^ Department of Emergency Medicine, LAC+USC Medical Center, Keck School of Medicine of University of Southern California, Los Angeles, California, USA.; ^5^ USC Gehr Family Center for Health Systems Science and Innovation, USC Keck Human Rights Clinic, Los Angeles, California, USA.

**Keywords:** asylum, qualitative, Migrant Protection Protocols, asylum seeker, social determinants of health, trauma informed care

## Abstract

**Introduction:** The Migrant Protection Protocols (MPP) required asylum seekers presenting to the U.S. southern border to wait in Mexico while seeking asylum. Currently, there is a lack of understanding of the MPP's potential harm to an already highly traumatized population. We sought to understand health impacts of this policy, including exposure to continued trauma.

**Methods:** The University of Southern California (USC)'s Keck Human Rights Clinic analyzed de-identified legal declarations and forensic medical affidavits of 11 asylum seekers subjected to MPP. A deductive, thematic analysis was performed to understand the health impact and traumas experienced, and instances of each subtheme were counted by utilizing content analysis methodology.

**Results:** Case analysis identified a total of 36 subthemes. Trauma subthemes included physical assault, psychological abuse, violence against family/friends, witnessed violence, sexual violence, and escalation. Perpetrator subthemes included gang, paramilitary, intimate partner, family, state, and unknown/other. Stress subthemes included despondency and social isolation. Security subthemes included reach of perpetrator, impunity of perpetrator, continued fear of persecution, fear of return, lack of safety, and reliance on strangers. Social determinants of health subthemes included tenuous housing, financial support, food insecurity, health care access, access to employment, and hazardous conditions. Psychological sequelae included anxiety, depressive, post-trauma, and suicidality; physical sequelae included dental, neurological, and dermatological sequelae.

**Conclusion:** The MPP caused harm among these 11 cases evaluated. Harm resulted from continued trauma, worsening social determinants of health, and continued presence of fear and insecurity. The MPP may increase the risk of re-traumatization as well as detract from asylum seekers' ability to heal from pre-migration trauma.

## Introduction

On January 25, 2019, The Department of Homeland Security implemented the Migrant Protection Protocols (MPP). The policy required that asylum seekers presenting to the southern border of the United States wait in Mexico during the determination of their asylum case. The policy applied to individuals who expressed fear of return to their country of origin if the individual “ha[d] been assessed not to be more likely than not to face persecution or torture in Mexico”.^1^ Exceptions to this policy included unaccompanied children, citizens or nationals of Mexico, individuals subjected to expedited removal, individuals with “known physical or mental health issues,” and returning legal permanent residents, among others.^1,2^ Implementation of MPP deviated from previous asylum policy, which historically had allowed asylum seekers to enter into the United States for the course of their asylum proceedings.^3,4^

During implementation of the MPP, 71,036 individuals were returned to Mexico, the majority of whom are seeking asylum from Honduras, Guatemala, Cuba, and El Salvador.^5^ The MPP was stopped by the Biden Administration on January 20, 2021.^6^ However, there is a dearth of medical literature documenting the health implications of asylum seekers' experiences while the policy was in effect.

Globally, asylum seekers experience a high degree of trauma, resulting in high rates of psychological sequelae, including major depressive disorder (MDD) and post-traumatic stress disorder (PTSD).^7–12^ Rates of PTSD, MDD, and anxiety are reported to be as high as 36%, 44%, and 40%, respectively, among refugee and asylum-seeking populations.^8^ These circumstances are similar for migrants in MPP from Central America, where the most common reason for flight is gang-related violence. Experiences of serious violence against family members, death threats, or threats of other violence are commonly reported.^13^

Advocates and journalists have documented lack of access to food, water, shelter, and means of communication among asylum seekers subjected to the MPP, as well as instances of kidnapping, robbery, and sexual assault.^14–16^ Reports highlight that, in practice, individuals with serious medical conditions have not been exempt from the policy, thus limiting access to appropriate medical care.^17^

These reports suggest that the asylum-seeking experience under the MPP are not congruent with global standards regarding the treatment of migrants and survivors of trauma. These include the guidelines set forth by the United Nations High Commissioner for Refugees, and the Substance Abuse and Mental Health Services Administration, which defines six fundamental principles of a trauma-informed approach^18,19^ This model maintains that recovery from past trauma cannot begin under situations of violence, instability, and fear.^19^

We aimed at understanding the health effects of MPP, both physical and psychological, as well as the social determinants of health that impact this vulnerable population, via a focused, descriptive review of legal documents generated on behalf of asylum seekers forced to remain in Mexico.

## Methods

### Data sources

The University of Southern California (USC) Keck Human Rights Clinic (KHRC) is a student-run organization that connects asylum seekers and their legal representation with volunteer clinicians trained to provide forensic medical examinations. The KHRC compiled a secure RedCap database containing deidentified legal declarations and forensic medical affidavits of 11 asylum seekers subject to the MPP who were evaluated by KHRC clinicians. A total of 24 documents were assessed. Each paired declaration and affidavit belonging to a single client were considered a document set. Client legal declarations included a narrative description of the client's experience and reason for seeking asylum, whereas medical affidavits included the client's history as well as the physical and psychological findings and assessment of the clinician. Each of the 11 cases was identified via the clinic's clinician evaluators and was confirmed to be subject to the MPP by the date of asylum claim and location of the client at the time of the evaluation. Legal representatives of all client documents involved were consulted regarding use of deidentified client documents, inclusion of quotes and provided review of the manuscript to ensure that clients were aware and consented to use, in addition to avoid any information that could imperil ongoing cases. This study was approved by both the USC Social Behavioral IRB and the Physicians for Human Rights Ethical Review Board.

### Thematic analysis

A deductive, thematic analysis of all documents was performed to understand traumas experienced by asylum seekers who were part of the MPP program. A thematic analysis methodology was selected to analyze the existing data set, as further purposive sampling could not be performed as required in a grounded theory methodology. After an initial sensitizing read of all documents, three investigators (R.F.P.L., M.C.S., T.W.S.) open coded all document sets in a line-by-line manner to generate preliminary codes of traumas, stressors, harms, and sequelae experienced by asylum seekers. We then reviewed the preliminary codes with a re-read of all documents, collapsing these into eight themes. We then iteratively refined these themes and subthemes to develop a final thematic map, including 8 themes and 36 subthemes. Each document was then coded by at least two investigators, and coding was compared. Disagreements in code application were settled by consensus. If consensus could not be achieved, the entire research team was consulted to reach a decision.

To ensure rigorous analysis and self-reflection, we employed a memoing method as codes were developed into themes, during thematic map development, and during refinement of the final thematic map. To improve trustworthiness, weekly meetings with the research team were held to discuss changes to codes. In addition, an iterative process of coding was used to develop themes and subthemes.

### Content analysis

In the thematic analysis, “stage of journey” was a major theme relating to traumas experienced by asylum seekers. After all documents were coded using the finalized thematic map, each document set was assessed for the presence or absence of each subtheme in the pre-migration, during migration, and post-asylum request periods. The post-asylum request time period has specific relevance to this analysis as representing instability of the asylum-seeking experience as a direct result of being subject to the MPP. The total number of each sub-theme was calculated at each time period.

## Results

**FIG. 1.**
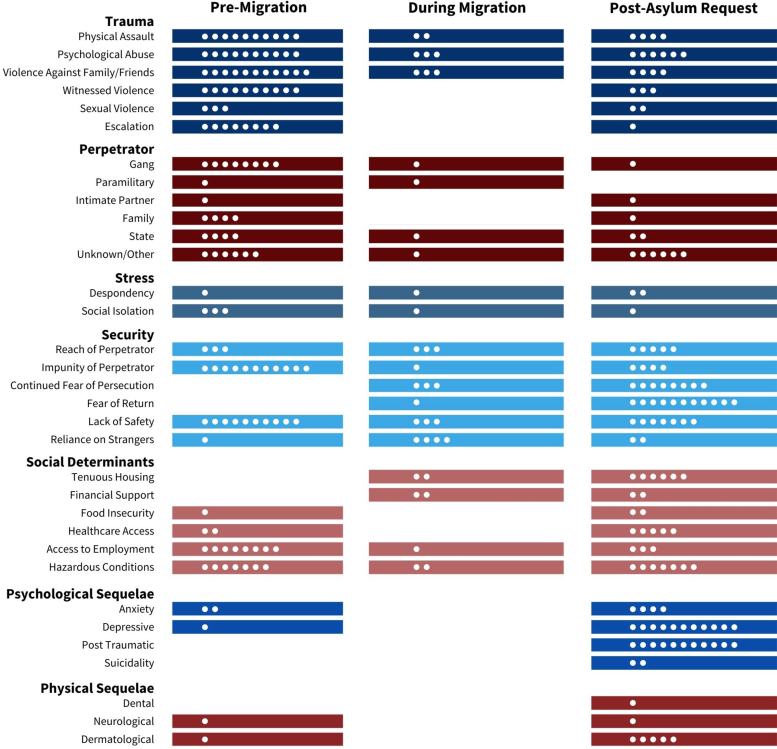
This figure illustrates the subthemes at different time points documented in each asylum seeker's declarations and affidavits (post-asylum request corresponds to the period the individual was under Migrant Protection Protocols). Each *white circle* represents one asylum seeker, with the maximum possible circles being 11 at each time point.

The final codebook from the thematic analysis included 8 themes and 36 subthemes (Appendix Table A1). The first theme identified was *stage of journey*, which included three subtheme periods: *pre-migration, during migration*, and *post-asylum request*. The *pre-migration* period was defined to end at the time an individual fled the country of origin. The *during migration* period then spanned from the time of departure until presentation at the U.S. border. The *post-asylum request* period was then defined to begin at presentation to the border and encompassed the MPP period. In addition, seven themes described the context of the ordeals experienced in the migration process: *trauma, perpetrator, stress, security, social determinants of health, psychological sequelae*, and *physical sequelae*. In the subsequent content analysis, the respective subthemes were mapped to each of the *stages of journey* (Fig. 1). Illustrative quotes for each subtheme are integrated later and listed in Table 1.

**Table 1.** De-Identified Quotes: De-Identified Quotes Extracted from Keck Human Rights Clinic Documents That Exemplify Each Subtheme

Themes	Sub theme	De-identified quote
Trauma	Physical assault	“They beat him with fists and feet over his entire body. He was struck in the face and his left eye. His front left incisor was avulsed. The assailants also repeatedly struck him with machetes.”
	Psychological abuse	“They also said they would let their daughter live, so that they could raise her as a ‘woman of the gang’.”
		“He threatened that if I said anything to my mom or if I report this to the police, he would do the same thing to my little brother.”
	Violence against family/friends	“[He] also learned that his partner was raped by a narcotrafficker. He reports that the narcotrafficker raped his wife and threatened to kill her. However, he states that the narcotrafficker told his wife that she was suffering this violence in an effort to break his will to live and to ‘hurt him in the worst way possible’.”
		“One day in 2018, members of [the gang] came to the firewood depot where my father worked as security guard, and demanded he give them free firewood. He refused. The gang members told him he would pay. They came back and shot him, and he died. I had to go to the morgue to identify his body. My siblings were too afraid that the gangs would identify them and target them if they went to the morgue.”
	Witnessed violence	“They were beating them and taking away their cell phones with which they were recording. There were a lot of people participating, and the police had received orders to capture each person being in ‘disarray.’ I put it this way because they were calling it disarray, even though we were all peaceful protesters. There were no commotions nor uproars like they were calling it, but since the police started to get violent, we defended ourselves with sticks, rocks, and whatever we could find. They began to shoot at us without caring how many lives they took.”
		“[He] witnessed [the gang] shoot a girl. [The gang] knew that he witnessed the shooting. [The gang] kidnapped him, bound him with rope, and took him to an alley. They said something like ‘do not open your mouth, do not say anything.’ He immediately thought he was going to be killed. They also said, ‘we will destroy your life if you report what you saw.’ They tortured him by beating him and fracturing his ankle and arm. He has scars from these beatings. He did not report the shooting to the police.”
	Sexual violence	“He physically assaulted her and attempted to rape her. He grabbed her arms, punched her, and shoved her to the ground.”… [She] was harassed again by the same man on two different occasions. He approached her both times with a gun and threatened to kill them.”
		“He raped me once or twice a week. I still didn't tell anyone because he repeatedly threatened me that if I told anyone about what he was doing to me, he would abuse and rape my brother and kill my family.”
	Escalation	“[He] told me that if he could not have me, no one could have me - escalating the previous death threats he had made to me, such as that I would wind up dead and no one would notice.”
		Trying to escape always made him even more angry, and said things like “if you run, it will only be worse.”
Perpetrator	Gang	“There was a girl in our neighborhood who got herself into trouble by getting into a relationship with someone from [the gang] and supposedly sharing secrets from [the opposing gang]. The [the opposing gang] kidnapped, raped, and killed her, and then they cut her into pieces. Just outside my door, I saw her head on a stick with a sign that said, ‘for being a snitch.’”
	Intimate partner	Shortly after I gave birth to our second son, He became violent and abusive toward me. He abused me physically and sexually. He would hold me down with his body weight on top of me and choke me until I could not breathe. He also raped me many times in our bed.
		He was also abusive toward our two children. He would hit our oldest son and attempted to hit our youngest son. I would hold our youngest son to protect him from him, who would hit me instead.
	Family	His attacks on me continued to become more and more violent, until I was sure that he would kill me if I continued to live with him. The worst assault in our home occurred in late 2017. He came home drunk from work. I had prepared a meal for him and was waiting at our dinner table. He grabbed me and threw me violently into the wall. My head hit the wall and I fell to the floor. I hit my head again on the floor as I fell, opening up a large cut on my scalp. He threw the food on the floor and left the house without concern for me as I lay bleeding on the floor from the cut on my head. For the next two to three days after the incident, I experienced dizziness, nausea and vomiting, and had trouble remembering details of the night of the incident. The cut on my head also bled periodically for three days. I remained in our house for two weeks recovering, since I did not have money to afford professional medical treatment and had to care for my two sons, including nursing my youngest son while I recovered. I have a large scar on my head to this day from his attack.
		“My mother was very harsh with my siblings and me. She beat us every time she thought that we disrespected her or did not do what she said. If my sister and I weren't getting along, or if I said something my mother didn't like, my mother hit me. She started hitting me when I was very young. From as early as I can remember, she hit me. She hit us with tree branches and belts - anything that was close. She also insulted us when she hit us. She told us we would never be anything in life and called us ‘stupid’ tonto burro.”
		“At one point, [partner's] stepfather tried to force me to have sex with him.”
		“When my grandparents got upset at me, they beat me with whatever they had. My grandfather used his belt to hit me a lot of the time. Sometimes he and my grandmother also used a chicote (whip) to hit me. They had it even though we didn't have any animals; they kept it to beat me when I did something wrong.”
	State	“I started to be investigated and harassed by [government agency]”
		“The policemen got out of the pickups and told us if we were picked up by them, they would make us disappear from the map for walking around causing commotion against the government. They already knew we participated in the marches that is why they were after us. They threatened to kill us and said we better watch our backs, and that this was not the end, one day they would eventually pick us up.”
	Paramilitary	“Two men who were from the [paramilitary group] threatened and harassed us for the simple fact that we were part of the opposition.”
		“[He] explains that a vigilante group known as the [paramilitary group] had for years terrorized his town with impunity. This included curfew establishment, restricting access to religious places of worship, military-type control of the city travel-ways, and extortion of civilians. Indigenous groups were particularly targeted, and the [paramilitary group] were known to force indigenous civilians to cut their hair, threaten those in indigenous garb, and execute people with indigenous inspired tattoos. The [paramilitary group] acted free from repose, as it was understood that the mayor of the town supplied the group with money and weapons.”
	Unknown	“It was about 3:00 p.m., at which point we were surprised by a vehicle from which armed men got out and came toward me. They grabbed me and I think they tried to kidnap me. I was trying to prevent them from grabbing me, so I fell to the ground with [redacted] in my arms, who was crying and screaming. These men took my cell phone and threatened me, saying that if I disclose the ‘information’ I had, they will kill me.”
Stress	Despondency	We arrived the day U.S. officials shot tear gas at the border. There was lots of chaos and harmed people at the shelter. The shelter was packed and living conditions were shocking. I questioned why I had come in the first place. I felt very disillusioned. I just wanted to be safe and protected.
	Social isolation	“They do not know anyone in Tijuana and have no one they can trust.”
Security	Reach of perpetrator	“They grabbed me, and I think they tried to kidnap me…I remember clearly that these men had accents [from my home country]. So, my fear was greater knowing that armed men like these could be a part of the [paramilitary groups] who are supported by [my country of origin's] government.”
		“I currently live in a shelter with my boys. It is unsafe for them to leave so they cannot attend school right now or leave too often. I know the gang at home has ties to [a different gang] in Mexico.”
	Continued fear of persecution	“They tried to kidnap me and threatened to kill me and again mentioned the ‘information’ I had. I started running as fast as I could with my child, and I ended up at a local [redacted] store. They also had an accent [from my home country], and they looked like the same men that threatened me [before].”
	Impunity of perpetrators	“My brother later filed a police report, but there was no investigation and nothing else was done.”
		“Despite providing evidence of the attack, he never heard again from the local authorities after filing this complaint.”
		“I never went to the police about the crimes and atrocities I saw around me because I knew it would not do any good and would just put me and my family in greater danger. I knew a teacher whose husband was killed by the gang. She filed a police report and they killed her.”
		I was too scared to report the incident to the police. In [my country of origin], if you report problems with the gangs to police, your family will start to have problems with the gangs. My neighbor's brother was murdered, and she went to the police. Then, [the gang] sent her a letter saying they didn't want to see her in the neighborhood anymore. Her family moved away. I don't know what happened to her and her family”
	Fear of return	“I fear for our lives. Especially, as a mother, I feel the responsibility of doing everything possible to protect my daughter from the harm I am sure she will suffer at the hands of [the gang] members, if we are deported to [my country of origin].”
		“I am very afraid of returning to [my country of origin]. I am scared that [they] may come back and try to hurt me again, and [they] might do the same thing to my little brother, too, just like [they] always threatened [they] would.”
		“My son and are not safe in Mexico and we will be persecuted if we return to [our country of origin].”
		“When I think about the possibility of being deported back to [my country of origin], I feel like a dead man. I could never return to [town of origin] and survive. The moment I would arrive, [the gang] would kill me. I have no doubt about it. I feel terrified and get panicked when I think about it. It's hard to explain, but it is a feeling that burns my soul and sucks out hope from my life.”
	Lack of safety	“I was afraid to spend time out in public or use public transportation because I was afraid that a gang member who knew [my perpetrator] would recognize me and kill me.”
		“I knew I wasn't safe because [the gang] has connections throughout the whole country and can find you anywhere. I knew they would kill me.”
	Reliance on strangers	“When we got off the bus in Mexicali, a man offered to drive us. He dropped us off at an unknown location and told me to walk in a certain direction.”
Social determinants of health	Tenuous housing	“After that, I started to move to different neighborhoods within Rosarito quite often. After all that I have been through, and knowing that groups like these operate freely, I have been on high alert and moved around so they cannot track me down easily. Whenever I saw a car with individuals that resembled those that attacked me, I moved to a different location. I have stayed in different apartments and hotels.”
		“The United States sent me to Tijuana after I was detained at the border, where I have stayed… I rent a room from a woman I met after being released. The room does not have a lock.”
	Hazardous conditions	“Her family initially stayed in a shelter, where they often heard gunshots and armed robbery immediately outside the confines of the shelter.”
		“Starting when I was a teenager, [the gang] began taking control of [town of origin], and it became very scary to live there. [The gang] controlled my hometown for a long time, and most of us lived in fear.”
	Access to employment	“They worked as live-in housekeepers but were asked to leave after a few months as relatives of the owners came to live and there was no longer any room for them.”
	Health care access	“[He] does describe experiences episodes of gout while in Matamoros. He has not been able to receive proper medical care for this due to the prohibitive cost.”
	Financial support	“Since the COVID-19 pandemic, their family has been unable to send money to help support them in Tijuana.”
	Food insecurity	“They have had a very unstable income source and, therefore, very insecure food and housing”
		“They were often without food, sometimes for four days at a time [in the shelter].”
Psychological sequelae	Anxiety	“Her guilt and feeling of hopelessness in protecting her three-year old son from the dangers in the city of Tijuana has brought her to the brink of a nervous breakdown and psychological exhaustion.”
	Depressive	“She has negative thoughts and feelings, including isolation, anhedonia, sleep disturbances, decreased interest in activities, and hopelessness.”
	Post traumatic	“There were many times when I thought I was going to die because of the beatings that I endured by the death squad in my town. I am traumatized by these things, and I constantly feel fear and panic at the thought or any reminder of them. And in addition to the attacks I experienced, I also witnessed horrifying things that the [paramilitary group] did to other people in my town that still haunt me to this day and give me nightmares.”
		“He also describes patterns of avoidance and hypervigilance: he rarely leaves his apartment. He is preoccupied with the notion that will be abducted by masked men, even in Tijuana. Additionally, he has difficulty concentrating and sleeping.”
Physical sequelae	Dermatologic	“[He] developed an eruptive skin condition that has not responded to any antibiotic ointment or topical steroids.”
		“[He] has multiple linear scars over his bilateral knees, right arm, and right hip. There are 5 well-healed linear lacerations to the right leg, mostly around the knee, which are in various angles, both parallel and perpendicular to the axis of the leg. These are consistent with his report of assault by machetes.”
	Neurologic	“She also reported consistent headaches for 3 months afterward, which she continues to occasionally have today.”
	Dental	“Tooth #9 is displaced laterally, consistent with the prior avulsion injury.”

### Trauma

Extensive trauma experienced in the pre-migration period provides the basis for fleeing the circumstances in the country of origin and are emblematic of the need to seek asylum. A variety of traumas was experienced and contextualized with six subthemes. Trauma persisted in the *during migration* and *post-asylum request* periods (see Fig. 1 for instances of subthemes in each period). The subtheme of *psychological abuse* was most common, followed by physical assault and *violence against family and friends*. One asylum seeker wrote:

“[T]hey beat me unconscious again. When I woke up, I was completely naked and bathed in blood. A couple of my neighbors were around me again, and they brought me to. My house. They told me they thought I was dead. I had a huge cut on my forehead, and I still have a scar from it today. I also had a bunch of marks and lashes on my back, as if they had whipped me over and over.”

### Perpetrator

*Perpetrator* as a theme described who persecuted the asylum seeker and included subthemes of *gang, paramilitary, intimate partner, family, state*, and *unknown/other*. These perpetrator figures were pervasive in the *pre-migration period* but also present in the *post-asylum request period.*

*Perpetrator* is well exemplified by an asylum seeker who described:

“[The gang] beat me, and kept demanding information about where I was from, where I lived, and why I was there. They told me I was in their territory and it was going to be ‘my last day’.” (Table 1)

### Security

The *security* theme described factors that informed asylum seekers' assessment and perception of their own safety. Subthemes included *reach of perpetrator, impunity of perpetrator, continued fear of persecution, fear of return, lack of safety*, *and reliance on strangers*. *Security* subthemes identified in the pre-migration period were *impunity of perpetrators, lack of safety, reach of perpetrator*, and *reliance on strangers*. In the *during migration* and *post-asylum request* periods, all *security* subthemes were present, including *continued fear of persecution* and *fear of return,* which is representative of the intense feelings of insecurity among this population during their journey and while forced to remain in Mexico. During the *post-asylum request* period, one asylum seeker reported:

“I received word that [they] were in Tijuana…[He] has seen me before and knows what I look like. I am in constant fear of being seen by [them] or other gang members in Mexico and being killed.”

### Stress

*Stress* described the psychological stressors (outside of mental illness) that created undue burdens on asylum seekers. Stress subthemes included *despondency* and *social isolation*, which were present in all periods compounding previous subthemes.

One clinician noted that their clients “do not know anyone in Tijuana and have no one they can trust.”

### Social determinants of health

Six subthemes of *social determinants of health* also arose from the data set. Subthemes identified in the *pre-migration* period were *access to employment, hazardous conditions, health care access*, and *food insecurity*. The identification of subthemes shifted in the *during migration* period, to *tenuous housing and financial support*, but excluded *food insecurity or health care access.* All *social determinants* subthemes were identified in the *post-asylum request* period*,* representing the most prominent period for social determinants of health subthemes (Fig. 1).

In order to meet subsistence needs, one asylum seeker *“[h]ad to take jobs to support [his family] that led him out of the city of Tijuana, and he had to leave his wife and [child] behind.”*

### Psychological sequelae and physical sequelae

Finally, the presence of *psychological and physical sequelae of trauma*, identified as major themes, illustrate the complex health burden of these asylum seekers. *Anxiety* and *depressive* subthemes were identified in the *pre-migration* period. None of the subthemes were identified during migration, whereas all (*anxiety, depressive, post-traumatic,* and *suicidality*) were identified *post-asylum request*. This suggests an increase in *psychological sequelae* among these 11 cases when comparing the *pre-migration* period with the *post-asylum request* period. All 11 cases identified *depressive* and *post-traumatic* subthemes *post-asylum request* (Fig. 1). One clinician described post-traumatic symptoms during the *post-asylum request* period, writing

“[S]he suffers from intrusive thoughts even now of these events almost every day. She has difficulty concentrating/sleeping, she is hypervigilant, and often feels an overwhelming sense of panic.”

*Physical sequelae* identified in the data set included *dental, dermatological, and neurological sequelae*. *Dermatological* and *neurological sequelae* were identified *pre-migration*. In the *post-asylum request* period, all subthemes were present in documentation by clinicians, in which they describe scars and headaches, among other physical findings (Fig. 1).

## Discussion

Globally, asylum seeker populations have experienced extensive pre-migration trauma and have been shown to have a high prevalence of PTSD and depression.^6–8,10,13^ In documents reviewed for these 11 individuals enrolled in MPP, pre-migration trauma was similarly pervasive. *Trauma* described among these 11 individuals included *physical*, *sexual*, and *psychological violence* as well as *witnessed violence* and *violence against family or friends*. Notably, these same *trauma* subthemes were identified in the data set in the *post-asylum request* period. This suggests that asylum seekers enrolled in MPP have experienced additional physical and psychological trauma after requesting asylum, thus prolonging trauma exposure and exacerbating their mental health.

This prolonged trauma exposure is important to understand in the context of the high proportion of these 11 individuals who screened positive for both PTSD and depression. *Post-traumatic* and *depressive* subthemes were identified in all 11 individuals included in this study. Although screening positive is not diagnostic for either condition, these results do align with the high prevalence documented in previous studies of asylum seeker populations.^6–8,10^ Importantly, cumulative exposure to stressful or traumatic events has been associated with increased risk for or clinical worsening of both PTSD and depression.^20–23^ Thus, these data suggest that MPP may lead to worsened mental health outcomes, via continued exposure to trauma among vulnerable and previously traumatized individuals.

As a result of both pre-migration trauma and compounded trauma after enrollment in MPP, asylum seekers may have experienced much from which they need to heal. To begin to heal from trauma, Substance Abuse and Mental Health Services Administration (SAMHSA's) safety principle of a trauma informed approach requires that individuals feel both “physically and psychologically safe.”^19^ Inconsistent with this principle, documents for these 11 individuals identified *security* and *social determinants of health* as persistent ordeals in the *post-asylum request* period. Subthemes of *tenuous housing*, *food insecurity*, and *hazardous conditions* and identification of *lack of safety*, *reach* and *impunity of perpetrators*, and *continued fear of persecution post-asylum* request suggest inadequate subsistence conditions and a lack of physical and psychological safety in this period. Peer support is also a central tenant of SAMHSA's trauma-informed approach.^18^ In our analysis, the subtheme of *social isolation* was also identified in the *post-asylum request* period, suggesting that lack of peer support may contribute to an environment incompatible with trauma recovery.

Similarly, guidelines for the international protection of refugees outlined by the United Nations High Commissioner for Refugees call for “the right to a standard of living adequate for health and well-being, including food, clothing, housing and medical and necessary social services.” Our data suggest that asylum seekers enrolled in MPP were not consistently afforded social welfare and health care as recommended by these guidelines. This, coupled with prolonged trauma exposure, requires consideration of the international human rights law principle of non-refoulement.^24^ The United Nations Office of the High Commissioner states that this principle “guarantees that no one should be returned to a country where they would face torture, cruel, inhuman or degrading treatment or punishment and other irreparable harm.”^25^ Further clarifying, “[t]his principle applies to all migrants at all times, irrespective of migration status.”^25^

Increased cumulative trauma exposure in the context of inadequate access to basic services creates an environment incompatible with healing from prior trauma. Importantly, not only does this pose a risk to the health of the individual asylum seeker, but also to their present and future family and community through the effects of intergenerational trauma.^26,27^

### Limitations

Our analysis is limited by the small sample size, as this population of asylum seekers is difficult to reach and understudied in the medical literature. Our dataset may be hypothesis generating for larger studies, but it also highlights critical health needs. A pre-existing dataset was used in this analysis and, therefore, only experiences documented in available declarations and affidavits were included. Thus, absence of subthemes in the available documents does not definitively represent absence of this experience in the population. Similarly, as these documents are written with the primary intent of documenting pre-migration factors leading to flight from country of origin and request for asylum, the traumas experienced in the “during migration” and post-asylum request periods may be underrepresented. To protect the safety and confidentiality of KHRC clients and prevent the possibility of tracing any information back to a single asylum seeker, all data were analyzed and presented in aggregate, which prevented any comparison between time periods for any one individual. Finally, all asylum seekers included in this study had access to legal representation for their asylum cases, and therefore both represent a small portion of the population of asylum seekers subject to MPP and a population whose attorneys determined might benefit from a medical or psychological evaluation.

## Conclusions

Our thematic analysis suggests that the institution of MPP may have been harmful to the physical and psychological health of enrolled asylum seekers. Prolonged trauma exposure in the context of inadequate access to subsistence resources may increase the risk of re-traumatization and detract from one's ability to heal from cumulative trauma. Thus, this policy and others, like it, should not be utilized in the care of asylum seekers due to the impact on their health and reverberations to the larger community. Further, more work is required to understand the extent of the damage resulting from MPP, especially at the population health level, such that appropriate reparative policies may be designed to heal these prior harms and prevent similar injurious U.S. immigration policies from being employed in the future.

### Implications for health equity

The MPP serve as an example of a policy with potentially detrimental health implications. This policy, in itself, should not be reinstated, nor should further policy be developed that might similarly endanger the health of vulnerable populations. To repair the damage done by this policy, the United States must consider supporting asylum seekers enrolled in this policy and enable them to safely seek asylum. We recommend that asylum seekers previously enrolled in MPP be allowed to enter and remain in the United States for the duration of their asylum proceedings, and that authorities consider how best to address medical and psychological needs exacerbated by the MPP policy. Not only might this mitigate long-term health harms for individuals enrolled in MPP but it may also prevent community health harms and propagation of intergenerational trauma.

## Abbreviations Used

KHRC: Keck Human Rights Clinic
MDD: major depressive disorder
MPP: Migrant Protection Protocols
SAMHSA: Substance Abuse and Mental Health Services Administration
USC: University of Southern California

## Appendix

**Table:** Table A1. Codebook: Codebook Describing the Themes and Subthemes Used in Thematic and Content Analysis

Themes	Subthemes	Definitions
Stage of journey	Pre-migration	Any events or experiences that occurred before departure from the client's home country.
	During migration	Any events or experiences that occurred after departure from the home country and before presenting to the U. S. border.
	Post-asylum request	Any events or experiences that occurred after presentation to the U. S. border for asylum and while in the Migrant Protection Protocols program.
Trauma	Physical assault	Any instance of physical assault against the client, including beating, use of blade, gunshot, burn, beating of feet (falanga), suspension, binding, asphyxiation, dental torture, water torture/drowning, positional torture, electric shock, etc.
	Psychological abuse	Any instance of psychological abuse against the client, including threats of violence, extortion, intentional humiliation, verbal abuse, devaluation, insults, threats of violence, threats of forced circumcision, mock execution, deprivation, forced marriages, forced pregnancy, covering, forced disclosure, conversion therapy.
	Violence against family/friends	Any instance of violence (physical or psychological) against family or friends of the client.
	Witnessed violence	Any instance of violence (physical or psychological) that the client witnessed.
	Sexual violence	Any instance of sexual violence against the client, including unwanted touching, penetration, forced circumcision, and other acts.
	Escalation	Any description of increasing frequency or severity of the trauma.
Perpetrator	Gang	Any reference to a gang member who is the perpetrator of the trauma to the client.
	Intimate partner	Any reference to an intimate partner who is the perpetrator of the trauma to the client.
	Family	Any reference to a family member who is the perpetrator of the trauma to the client.
	State	Any reference to a uniformed or non-uniformed person associated with the state.
	Paramilitary	Any reference to a non-state military organization.
	Unknown	Any reference to a perpetrator of the trauma that falls outside of the previously defined perpetrator subthemes or a perpetrator who is unknown to the client.
Stress	Despondency	Any reference to lack or loss of hope or loss of motivation. Any reference to the inability to continue the client's asylum claim.
	Social isolation	Any reference to lack of access to community, family, friends, or other form of social support for the client's needs.
Security	Reach of perpetrator	Any reference to fear of additional harm perpetrated by a perpetrator from the home country or associates of this perpetrator.
	Continued fear of persecution	Any reference to fear of additional harm or trauma.
	Impunity of perpetrators	Any reference to the belief or experience that perpetrators are not held accountable for, including experience reporting a crime that was not addressed by the authorities and the decision not to report based on perceived danger or presumed inaction on the part of the authorities.
	Fear of return	Any reference to fear of returning to the home country, fear of individuals, organizations, or experiences in the home country.
	Lack of safety	Any reference to feelings or experiences of being unsafe physically or psychologically.
	Reliance on strangers	Any reference to the need for assistance from unknown others or experiences relying on unknown others.
Social determinants of health	Tenuous housing	Any reference to instability of housing of lack of certainty with regard to housing.
	Hazardous conditions	Any reference to living conditions that may be harmful to health or well-being, physical or psychological.
	Access to employment	Any reference to the process of seeking employment, stability of employment, or conditions of employment.
	Health care access	Any reference to health care access or lack thereof, experiences of seeking care, or experiences requiring care regardless of whether it was sought.
	Financial support	Any reference to current means of financial support, including funds provided by family, friends, or community in home country or within the United States.
	Food insecurity	Any reference to the process of feeding oneself or one's family, barriers to access, and assistance from individuals or organizations.
Psychological sequelae	Anxiety	Any clinician reference to symptoms of anxiety.
	Depressive	Any clinician reference to poor mood, anhedonia, a motivation, poor energy, fatigue, poor concentration, appetite change, weight change, sleep change, guilt, hopelessness, worthlessness.
	Post traumatic	Any clinician reference to alterations in arousal, avoidance, negative cognitions, re-experiencing, dissociation, re-traumatization.
	Suicidality	Any clinician reference to thoughts of death, passive death wish, plan to commit suicide, attempts.
Physical sequelae	Dermatological	Any clinician reference to physical sequelae of trauma due to blunt injury, penetrating injury, laceration, burn, scarring, belt, or medical care.
	Neurological	Any clinician reference to physical sequelae of trauma that is neurological (traumatic brain injury, headaches, memory or other cognitive deficits, chronic pain).
	Dental	Any clinician reference to physical sequelae of trauma due to dental harm.
